# Assessing the necessity of a family of genes that encode small proteins in *Dictyostelium discoideum* development

**DOI:** 10.17912/micropub.biology.000490

**Published:** 2021-10-28

**Authors:** Yumei Wu, Felicia N Williams, K Matthew Scaglione

**Affiliations:** 1 Department of Molecular Genetics and Microbiology, Duke University; 2 Department of Neurology, Duke University; 3 Duke Center for Neurodegeneration and Neurotherapeutics, Duke University

## Abstract

*Dictyostelium discoideum’s* genome encodes for a large class of small proteins that are developmentally regulated. We deleted six of the genes that encode these proteins to determine if they play an essential role in *Dictyostelium discoideum* development. Deletion of these genes had no significant effect on *Dictyostelium discoideum* development. These results suggest that the selected genes do not play an essential role in *Dictyostelium discoideum* development.

**Figure 1. Deletion of a subset of genes that are developmentally regulated and encode for small proteins does not inhibit Dictyostelium discoideum development:**
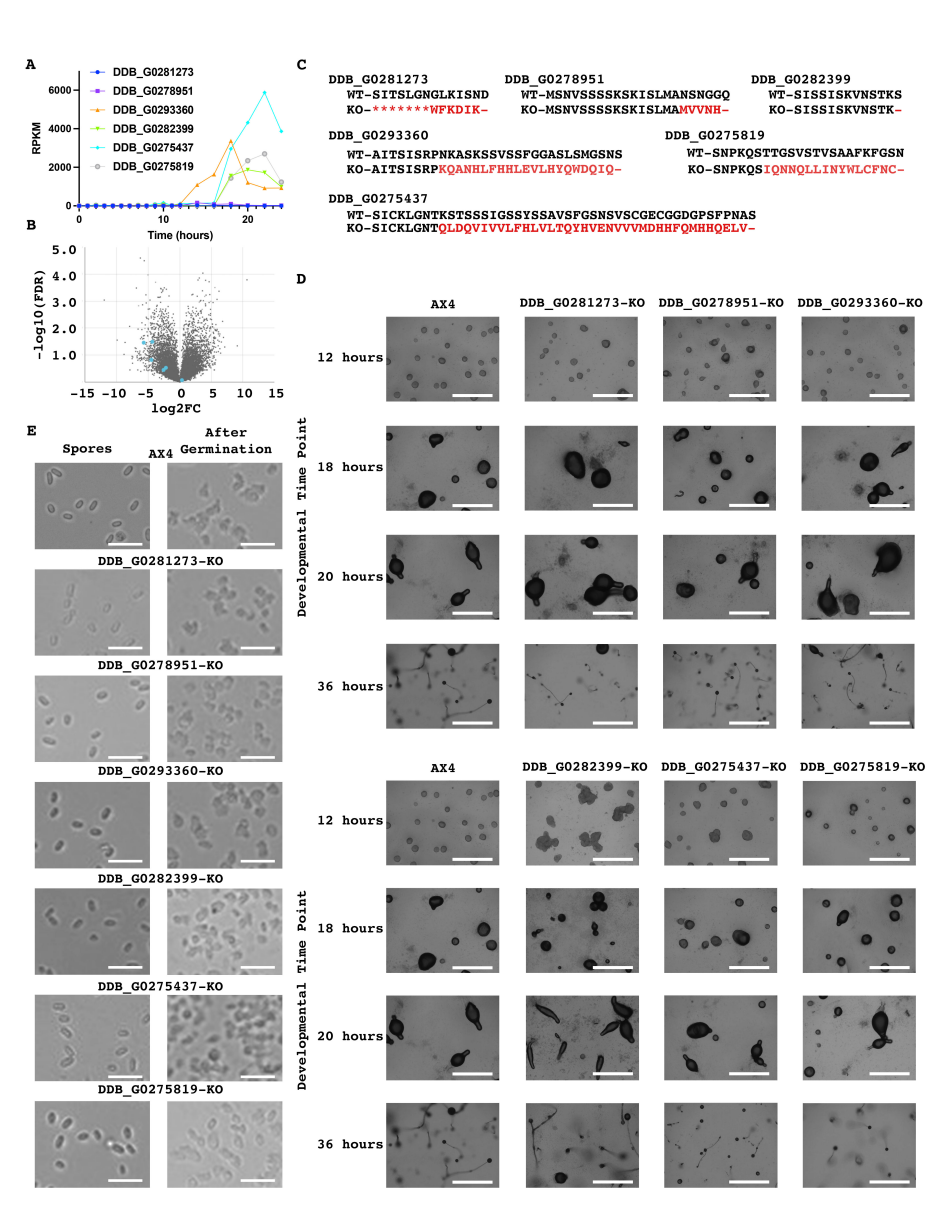
(A) Analysis of the expression pattern of selected genes during *Dictyostelium discoideum* development. (B) Five of the six genes selected are preferentially expressed in pre-stalk cells. (C) We generated knockout *Dictyostelium discoideum* strains using CRISPR/Cas9. The amino acid sequence of wild-type and mutant proteins are shown with mutated residues in red, (-) indicating a premature stop codon, and (*) indicating deleted residues. (D) Wild-type (AX4) and mutant strains were developed and imaged at the time points indicated. No difference in *Dictyostelium discoideum* development was evident when comparing the knockout strains to wild-type (n≥3; scale bars=1,000 microns). A single AX4 set of images is shown for comparison against all mutants. (E) Spores from wild-type and mutant strains were isolated and germinated. All strains formed viable spores that germinated into amoeba (n=4; scale bars=5 microns).

## Description

*Dictyostelium discoideum* are single cellular amoeba that are bacterivores, however, when starved, they undergo a developmental process and form a multicellular fruiting body. The fruiting body is composed of stalk cells that form a rigid platform to elevate a spore-filled sorus. This allows for the spores to be distributed to a new environment where they can germinate in search of a bacterial food source. This developmental cycle makes *Dictyostelium discoideum* a powerful model organism for investigating several cellular pathways including cell fate and tissue development.

In addition to having a unique developmental cycle, *Dictyostelium discoideum* is also interesting because its proteome encodes a massive amount of homopolymeric amino acid tracts (Eichinger *et al.* 2005). This includes homopolymeric amino acid tracts of every amino acid except tryptophan, including nearly 2,500 polyglutamine tracts (Eichinger *et al.* 2005). This is interesting because expanded polyglutamine tracts cause a class of nine neurodegenerative diseases in humans. We and others previously have demonstrated that *Dictyostelium discoideum* is naturally resistant to polyglutamine aggregation (Malinovska *et al.* 2015; Santarriaga *et al.* 2015). Further work from our lab identified a gene that encodes for a small 88 amino acid protein that imparts resistance to polyglutamine aggregation in *Dictyostelium discoideum* and in other model systems (Santarriaga *et al.* 2018). Using Panther Gene Ontology, we found that it was a member of a large class of genes that encode for small proteins. A subset of the genes that encode these proteins were previously identified as being developmentally regulated and the addition of antibodies against a subclass of these proteins prevented development (Vicente *et al.* 2008). Here we selected a subset of these genes to determine if they were essential for *Dictyostelium discoideum* development. Genes that have different time points of peak expression during development were chosen for analysis. Using CRISPR-Cas9 we developed knockout strains of the selected genes and demonstrated that they are not essential for *Dictyostelium discoideum* development.

To determine if these genes are developmentally regulated we utilized dictyExpress and found that these genes’ expression levels are developmentally regulated (Figure 1a). Further employing dictyExpress we found that five of the six were preferentially expressed in pre-stalk cells with one being preferentially expressed in pre-spore cells (Figure 1b) (Stajdohar *et al.* 2017). Utilizing CRISPR-Cas9, we developed knockout strains for these six genes (Figure 1c). We next developed wild-type AX4 cells alongside our six knockout strains and imaged them at specific time points. Similar to wild-type cells, all six mutant strains developed at a comparable rate and successfully formed fruiting bodies (Figure 1d). In addition, we assessed spore formation and germination and found that there were no obvious defects in these mutants (Figure 1e). Together our data indicate that these six genes are not essential for *Dictyostelium discoideum* development.

While we did not identify any developmental phenotype with these knockout strains it is possible that collectively this family of genes plays an important role during *Dictyostelium discoideum* development. This may occur as significant genetic redundancy among this group of genes and several of them have similar developmental expression patterns. This raises the possibility that the deletion of any one of these genes may be insufficient to result in a phenotype.

## Methods

*General Culture*

*Dictyostelium* cells were maintained in HL5 media at concentrations between 1-4 x 10^6^ cells per mL to keep the cells in mid-log phase. Cells were grown in flasks at 22°C with gentle shaking prior to gene knockout.

*Gene Knockout by CRISPR-Cas9*

Gene knockout was conducted by CRISPR-Cas9 as previously described (Sekine *et al.* 2018). CRISPR single guide RNA (sgRNA) sequences were designed using the CRISPR RGEN Tools Cas-designer and checked for off-target sites using the CRISPR RGEN Tools Cas-OFFinder (Table 1) (Bae *et al.* 2014; Sekine *et al.* 2018). sgRNAs were cloned into the pTM1285 vector (acquired from the Dictyostelium Stock Center) using BpiI (Sekine *et al.* 2018).

To conduct gene knockout, AX4 wild-type *Dictyostelium* *discoideum* cells were electroporated with each pTM1285-sgRNA construct using the standard protocol described on Dictybase (Pang *et al.* 1999). Approximately 5 x 10^6^ cells were pelleted by centrifugation at 500g for five minutes and then washed two times with H50 buffer. Cells were resuspended in 100µL of H50 buffer and 10µg of pTM1285-sgRNA plasmid DNA. The cell-DNA mixture was then transferred to a pre-chilled 0.1cm cuvette. Cells were electroporated at 850V for two pulses with a time constant of 0.6 msec and five seconds between pulses. Following electroporation, the cells were allowed to rest on ice for five minutes before being transferred to a 10cm Petri dish containing 10mL of HL5. G418 was added to the plates 8-16 hours after electroporation to a final concentration of 10µg/mL. The cells were collected 1-3 days after G418 addition and plated on SM agar plates with *Klebsiella aerogenes* bacterial lawns. Clonal isolates were picked and transferred to 96-well plates containing HL5 following the formation of plaques approximately four to six days after plating.

Once sufficient growth was observed in the 96-well plates, cells were transferred to larger 24-well plates. Upon reaching a minimum concentration of 2 x 10^4^ cells/mL, cells were collected from each well and pelleted by centrifugation at 500g for five minutes. Media was removed from cell pellets before cells were lysed with 4 volumes of lysis buffer prior to heat inactivation of proteinase K at 95°C for one minute (Charette and Cosson 2004). To screen for the generation of indels by CRISPR, we PCR-amplified a small region surrounding the sgRNA target site for each gene and ran the amplified DNA on a 4.5% agarose gel. Clonal isolates whose PCR products appeared to differ in size when compared to wild-type were cloned into the pCR4-TOPO vector using the TOPO TA Cloning Kit for Sequencing (ThermoFisher, cat. #K457502) and sent for Sanger sequencing using the M13 reverse sequencing primer. Mutations that resulted in a frameshift mutation that was not a multiple of three were considered to be knockouts.

Primers used for screening are shown in Table 3. Ideally, the amplified region would cover the 100bp flanking the cut site on either side to produce an amplicon approximately 200bp in length. However, in locations where PCR primers are not easily designed, amplicons of up to 400bp are also suitable to assess indel generation.

*Development*

To induce multicellular development of the *Dictyostelium* cells, 1 x 10^8^ cells were collected by centrifugation at 500g for five minutes. Cells were washed three times with cold development buffer and then resuspended in 350uL of development buffer. The cells were then spread on KK2 agar plates. These plates were wrapped in damp paper towels and plastic wrap before being incubated at 22°C.

*Spore Preparation and Germination*

Wild-type (AX4) or mutant *Dictyostelium* strains were developed for two to three days prior to spore isolation. Spores were isolated by scraping fruiting bodies into a 50mL conical with 30mL of cold HL5. The tube was then vortexed for five to ten seconds three times prior to centrifugation at 500g for five minutes. The supernatant was discarded, and the pellet for one 10cm dish was resuspended in 2mL HL5 with 10% DMSO by vortexing. Spores were frozen by placing 1mL aliquots at -20°C for one hour prior to transferring to -80°C. For germination one 1mL tube of spores was thawed at 37°C then the contents were counted and 1X10^6^ cells were transferred to a 24-well plate containing 1mL HL5 medium. Spores were allowed to settle for one hour prior to removal and replacement of HL5 to remove DMSO. Spores were allowed to germinate for 24 hours prior to imaging.

## Reagents

HL5 medium (1L):

· To 1L ddH_2_O, add:

o 17.8g proteose peptone

o 7.2g yeast extract

o 0.54g Na_2_HPO_4_

o 0.4g KH_2_PO_4_

o 130ul B12/folic acid mix

· Autoclave

· Prior to use, add the following (filter sterilized):

o 20mL of 50% glucose

o Streptomycin (300 µg/mL)

o Carbenicillin (100 µg/mL)

B12/folic acid mix:

· To 100ul ddH_2_O, add:

o 5mg B12

o 200mg folic acid

· Adjust pH to 6.5 and filter sterilize. Store at -20°C.

10X KK2 buffer (1L):

· To 1L ddH_2_O, add

o 22g KH_2_PO_4_

o 7.0g K_2_HPO_4_

· Filter sterilize. Store at 4°C.

· Use at 1X concentration by diluting with sterile ddH_2_O.

H50 electroporation buffer:

· In ddH_2_O, add the following to the specified concentrations:

o 20mM HEPES

o 50mM KCl

o 10mM NaCl

o 1mM MgSO_4­_

o 5mM NaHCO_3_

o 1mM NaH_2­_PO_4_

· Autoclave and store at 4°C

Lysis buffer:

· In ddH_2_O, add the following to the specified concentrations:

o 50mM KCl

o 10mM Tris pH 8.3

o 2.5mM MgCl_2_

o 0.45% NP40 (or IGEPAL CA360)

o 0.45% Tween 20

· Filter sterilize and store at room temperature.

· Prior to use, add 1ul of 20µg/mL Proteinase K for every 25ul of lysis buffer.

KK2 agar (1L):

· To 1L of 1X KK2 buffer, add:

o 15g agar

· Autoclave and pour about 20mL per 10cm petri dish.

Development buffer (DB; 1L):

· To 600mL ddH_2_O, add:

o 200mL of 5X phosphate solution

o 100mL 10X CaCl_2_ solution

o 100mL 10X MgCl_2_ solution

· Filter sterilize. Store at 4°C.

5X phosphate solution:

· In ddH_2_O, add the following to the specified concentrations:

o 25mM Na_2_HPO_4_

o 25mM KH_2_PO_4_

· Adjust pH to 6.5 and filter sterilize.

10X CaCl_2_ solution:

· In ddH_2_O, prepare a 10mM CaCl_2_ solution.

· Filter sterilize.

100mL 10X MgCl_2_ solution:

· In ddH_2_O, prepare a 20mM MgCl_2_ solution.

· Filter sterilize.

Table 1: Primers used for sgRNA cloning into pTM1285.

**Table:** 

Target gene	Primer	Sequence (5´ to 3´)
*DDB_G0281273*	sgRNA-1 sense(+)	AGCATCAATCACAAGTTTAGGTAA
	sgRNA-1 antisense	AAACTTACCTAAACTTGTGATTGA
*DDB_G0278951*	sgRNA-1 sense(+)	AGCATCTTTAATGGCAAATTCCAA
	sgRNA-1 antisense	AAACTTGGAATTTGCCATTAAAGA
*DDB_G0293360*	sgRNA-1 sense(-)	AGCAGATGATTTGCTTGCTTTGTT
	sgRNA-1 antisense	AAACAACAAAGCAAGCAAATCATC
*DDB_G0282399*	sgRNA-1 sense(+)	AGCAATTATCAATCAAGGTTGATT
	sgRNA-1 antisense	AAACAATCAACCTTGATTGATAAT
*DDB_G0275437*	sgRNA-1 sense(-)	AGCAAATTGAGGATGAAGTTGATT
	sgRNA-1 antisense	AAACAATCAACTTCATCCTCAATT
*DDB_G0275819*	sgRNA-1 sense(+)	AGCAAATCCAAAACAATCAACTAC
	sgRNA-1 antisense	AAACGTAGTTGATTGTTTTGGATT

Table 2: Strains used in this publication. All strains are available from the Dictyostelium Stock Center.

**Table:** 

Wild-type/Knockout Dictyostelium Discoideum strains(DDB#)	Knockout gene	Strain summary
AX4	N/A	Wild-type
DDB_G0281273-KO	*DDB_G0281273*	72bp deletion
DDB_G0278951-KO	*DDB_G0278951*	7bp deletion
DDB_G0293360-KO	*DDB_G0293360*	4bp deletion
DDB_G0282399-KO	*DDB_G0282399*	28bp deletion
DDB_G0275437-KO	*DDB_G0275437*	19bp deletion
DDB_G0275819-KO	*DDB_G0275819*	25bp insertion

Table 3: Primers used for screening of indel generation by CRISPR-Cas9.

**Table:** 

Knockout gene(Target gene)	Screening/Sequencingprimers	Sequence (5´ to 3´)
*DDB_G0281273*	Screening-Rv	ACAGATAAAATGGCAATTATTGG
	Screening-Fw	GTTTTTGATGGTGGTCCTCC
*DDB_G0278951*	Screening-Rv	TTTAGTATGAGCAACACCTACTGC
	Screening-Fw	ATGATACATATTAAAAAACTAAATACTGATG
*DDB_G0293360*	Screening-Rv	ACAGAAAACCATCAAATCAAACATC
	Screening-Fw	CACCACCAACAACAACACCC
*DDB_G0282399*	Screening-Rv	ATCAAATAATTAAAAAATGTCTCTCTTTGG
	Screening-Fw	CCAGCTTTATTTAAACTCTCAGC
*DDB_G0275437*	Screening-Rv	ATACTAGTTTGTTTGGTTTATTAATG
	Screening-Fw	AAACAATCAACTTCATCCTCAATT
*DDB_G0275819*	Screening-Rv	CCAAGTAAACCTTTATTTCCTCC
	Screening-Fw	ATACCAATTTAAAATGACAATCATTGG
